# Connectivity alteration in thalamic nuclei and default mode network-related area in memory processes in mesial temporal lobe epilepsy using magnetoencephalography

**DOI:** 10.1038/s41598-023-37834-2

**Published:** 2023-06-30

**Authors:** Tomotaka Ishizaki, Satoshi Maesawa, Daisuke Nakatsubo, Hiroyuki Yamamoto, Jun Torii, Manabu Mutoh, Jun Natsume, Minoru Hoshiyama, Ryuta Saito

**Affiliations:** 1 Department of Neurosurgery, Nagoya University Graduate School of Medicine, 65 Tsurumai, Showa, Nagoya, Aichi 466-8550 Japan; 2 Brain and Mind Research Center, Nagoya University, Nagoya, Aichi Japan; 3 Department of Pediatrics, Nagoya University Graduate School of Medicine, Nagoya, Aichi Japan

**Keywords:** Epilepsy, Brain

## Abstract

This work aimed to investigate the involvement of the thalamic nuclei in mesial temporal lobe epilepsy (MTLE) and identify the influence of interictal epileptic discharges on the neural basis of memory processing by evaluating the functional connectivity (FC) between the thalamic nuclei and default mode network-related area (DMNRA) using magnetoencephalography. Preoperative datasets of nine patients with MTLE with seizure-free status after surgery and those of nine healthy controls were analyzed. The FC between the thalamic nuclei (anterior nucleus [ANT], mediodorsal nucleus [MD], intralaminar nuclei [IL]), hippocampus, and DMNRA was examined for each of the resting, pre-spike, spike, and post-spike periods in the delta to ripple bands using magnetoencephalography. The FC between the ANT, MD, hippocampus, and medial prefrontal cortex increased in the gamma to ripple bands, whereas the FC between the ANT, IL, and DMNRA decreased in the delta to beta bands, compared with that of the healthy controls at rest. Compared with the rest period, the pre-spike period had significantly decreased FC between the ANT, MD, and DMNRA in the ripple band. Different FC changes between the thalamic nuclei, hippocampus, and DMNRA of specific connections in a particular band may reflect impairment or compensation in the memory processes.

## Introduction

Patients with epilepsy often experience chronic cognitive dysfunction. In addition, interictal epileptic discharges (IEDs) can cause transitory cognitive impairment^1^. These cognitive dysfunctions result from reduced functional connectivity (FC) due to the accumulation of transient damage to normal networks, such as the default mode network (DMN), during IED propagation through abnormal epileptic networks^2,3^. For example, a resting-state functional magnetic resonance imaging (fMRI) analysis conducted on patients with temporal lobe epilepsy (TLE) revealed a reduced FC between the DMN and hippocampus on the affected side^2^. Furthermore, electroencephalography (EEG)-fMRI studies have revealed that IEDs decrease the FC of various DMN parts and induce changes in neural activity before and after IEDs^3,4^. These findings suggest that changes in FC between the hippocampal memory networks and DMN in the resting state and during IEDs contribute to cognitive dysfunction, including memory and attention, in patients with TLE^3^.

**Figure 1:**
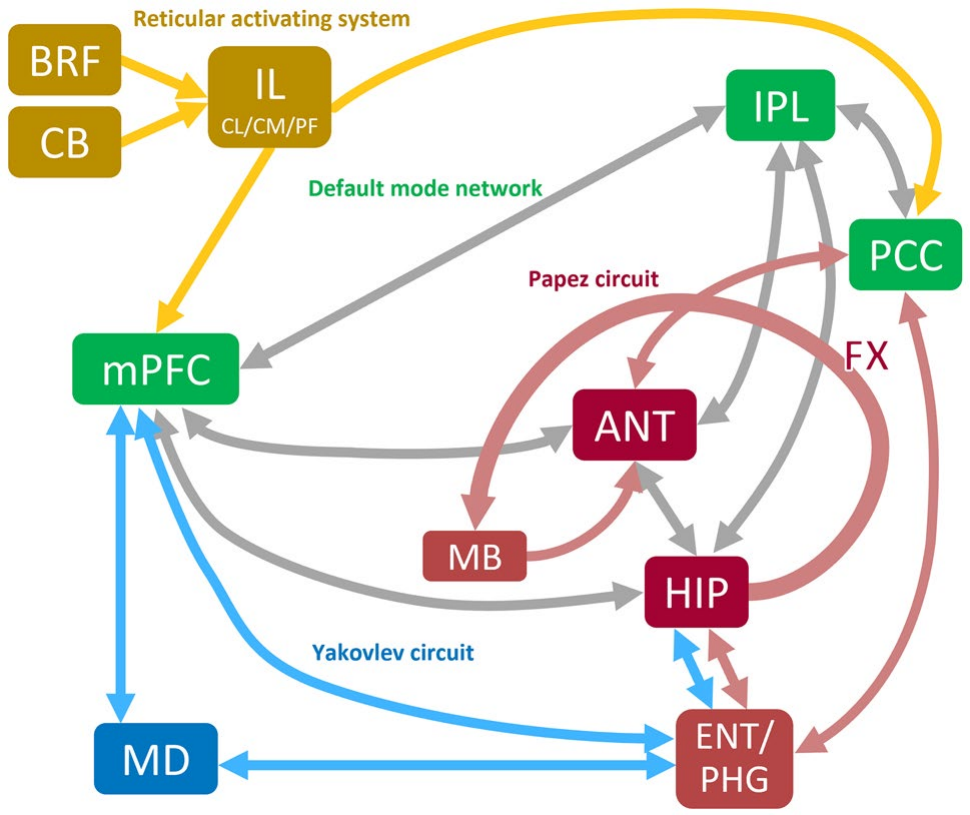
Thalamocortical networks involved in the memory process. The thalamocortical networks involved in the memory process include the Papez circuit (red arrow), Yakovlev circuit (blue arrow), and reticular activating system (yellow arrow). Default mode network core systems are indicated by green boxes. Abbreviations: BRF, brainstem reticular formation; CB, cerebellum; IL, intralaminar nuclei of thalamus; CL, central lateral nucleus; CM, centromedian nucleus; PF, parafascicular nucleus; IPL, inferior parietal lobule; PCC, posterior cingulate cortex; mPFC, medial prefrontal cortex; ANT, anterior nucleus; FX, fornix; MB, mammillary body; HIP, hippocampus; MD, mediodorsal nucleus; ENT, entorhinal cortex; PHG, parahippocampal gyrus.

The cerebral cortices and subcortical structures, including the thalamus, could be strongly involved in epileptic seizures^5^. In particular, the anterior nucleus (ANT), mediodorsal nucleus (MD), and intralaminar nuclei (IL) of the thalamus are important hubs in the three memory processes, encoding, retention, and retrieval, which receive inputs from the cortical and subcortical areas and project to specific cortical areas^6^. The connections between the thalamic nuclei, ANT, MD, and IL and the brain regions involved in the memory process are understood as shown in Fig. 1.

The ANT selects and coordinates information in processes for encoding and retrieval^7,8^. The ANT is connected to the hippocampus and cortical areas of the DMN core system through a network called the Papez circuit, and the coupling between the ANT and those cortical areas with specific frequency bands is important for the memory process^7^. The MD is connected to the medial prefrontal cortex (mPFC) and amygdala through the Yakovlev circuit network and to the hippocampus via the entorhinal cortex, and it plays a role in selecting and coordinating strategies for retrieval in the memory process^8,9^. The IL receive input from the brainstem reticular formation and connect via other thalamic nuclei to cortical areas necessary for memory processes. Moreover, they are responsible for activating the regions or circuits necessary to perform encoding and retrieval processes^8,10^.

Neural activity coupling within specific frequency bands are important in the thalamic nuclei, hippocampus, and DMN networks, all of which are involved in memory processes^7^. The coupling of neural activity across multiple frequency bands is important in the hippocampal-based memory process, particularly activities in the theta, gamma, and ripple bands^11^. In memory consolidation, neural activity in the ripple band of the hippocampus extensively activates the cortex and inhibits the diencephalon, midbrain, and brainstem^12^. Neural activity within specific frequency bands also involves abnormal network development in epilepsy. For example, in a rat model of epilepsy induced by kainic acid administration, abnormal ripple-band neural activity was observed in remote areas, such as the prefrontal cortex, thalamus, and contralateral hippocampus. This finding indicates that the acquisition of epileptogenicity is involved in the acquisition of a large abnormal network strongly associated with neural activity within the ripple band^13^. Hence, the abnormal network and memory impairment in patients with epilepsy may involve specific networks and neural activity within specific frequency bands. Thus, FC in the brain of patients with epilepsy changes at rest and during IEDs. These changes in FC reflect alterations in normal and abnormal network activities, which may occur within specific frequency bands. In other words, analyzing FC changes in each frequency band in patients with mesial TLE (MTLE) may provide information on changes in neural activity within networks specific to patients with MTLE, particularly those associated with memory impairment.

Based on the findings of these studies, we hypothesized that the memory impairment that occurs in MTLE is caused by the formation of abnormal networks among three thalamic nuclei (ANT, MD, and IL), which are involved in memory processes and the DMN, and the disruption of normal networks due to repeated epileptic discharges via abnormal networks. As this abnormal network due to epilepsy could be coupled with the activity of IEDs, we hypothesized that the increase in FC before and after IEDs would indicate the abnormal network activity and the activity of the normal network disturbed by the abnormal network would be represented by FC decreases before and after IEDs. We further hypothesized that the increase and decrease in FC compared with that in healthy controls would indicate chronic compensation and dysfunction of memory, and these FC changes would differ in each frequency band.

In this study, the following experiments were conducted to investigate the involvement of the thalamic nuclei in memory disturbance in MTLE. Activity between the thalamic nucleus and hippocampus of the affected side and the DMN-related area (DMNRA), including the mPFC, inferior parietal lobule (IPL), and posterior cingulate cortex (PCC), was analyzed for (1) temporal FC changes caused by IEDs and (2) FC changes in MTLE at the interictal state compared with the FC of healthy controls for each frequency band using magnetoencephalography (MEG). Although recording neural activity in deep structures using MEG remains controversial, the authors attempted to evaluate it using a combination of distributed source analysis and volume head modeling with statistical analysis^14^.

This analysis predicted that FC changes between thalamic nuclei, hippocampus, and DMNRA during IEDs and at rest would exhibit different patterns in different frequency bands, suggesting neural activity in the networks involved in memory impairment in patients with MTLE.

## Results

### Clinical profiles

Nine patients were included in the final analysis, and Tables 1 and 2 present their clinical profiles. All patients were followed up for more than 24 months after surgery (mean, 53.3; range, 24–85 months) and had a good surgical outcome at Engel class I. The left hemisphere was dominant in all patients with MTLE, and the epileptogenic zone was identified in the left in six cases. The Wechsler Memory Scale-Revised (WMS-R) results showed that eight patients had verbal memory decline (index below 85), while the visual memory of nine patients and the attention and concentration of eight patients were maintained above average. Multiple comparisons (Tukey’s method) between the WMS-R sub-items showed that the participants had significantly lower scores in general and verbal memory than in visual memory (general vs. visual: *P* = 0.010; verbal vs. visual: *P* < 0.001) and attention/concentration (general vs. attention/concentration: *P* = 0.019; verbal vs. attention/concentration: *P* < 0.001). Clinical profiles of nine healthy controls are presented in Supplementary Table S1.

**Table 1:** Clinical profiles.

Case #	Sex/age	Epilepsy type	Seizure type and semiology	Dominant side	Surgery	Resection area	Pathology	Engel class	Follow-up (months)
1	F/27	L-MTLE	Olfactory aura, R-hand dystonic posture, LOC	L	L-SAH	L-Hip, Amy, PHG, Un	HS	Ia	85
2	M/62	L-MTLE	R-arm tonic seizure, LOC	L	L-ATL	L-Hip, Amy, PHG, FuG, Un, TP, aSTG, aMTG, aITG	HS	Ia	73
3	M/44	L-MTLE	Oral and hand automatism, head and trunk version, LOC, FBTCS	L	L-SAH	L-Hip, Amy, PHG, Un	HS	Ic	75
4	M/40	R-MTLE	L-facial spasm, head and trunk version, LOC, FBTCS	L	R-ATL	R-Hip, Amy, PHG, FuG, Un, TP, aSTG, aMTG, aITG	Gliosis	Ic	71
5	F/44	R-MTLE	LOC, tonic posture	L	R-SAH	R-Hip, Amy, PHG, Un	FCD type IIb	Ia	44
6	M/30	L-MTLE	LOC, FBTCS	L	L-SAH	L-Hip, Amy, PHG, Un	FCD type IIb	Ia	43
7	M/19	L-MTLE	Oral and hand automatism, LOC	L	L-ATL	L-Hip, Amy, PHG, FuG, Un, TP, aSTG, aMTG, aITG	HS	Ia	41
8	F/19	L-MTLE	Hand automatism, LOC	L	L-SAH	L-Hip, Amy, PHG, Un	FCD type Iib	Ia	24
9	M/56	R-MTLE	LOC, FBTCS	L	R-ATL	R-Hip, Amy, PHG, FuG, Un, TP, aSTG, aMTG, aITG	HS	Ia	24

*F* female, *M* male, *MTLE* mesial temporal lobe epilepsy, *LOC* loss of consciousness, *FBTCS* focal to bilateral tonic–clonic seizure, *SAH* selective amygdalohippocampectomy, *ATL* anterior temporal lobectomy, *Hip* hippocampus, *Amy* amygdala, *PHG* parahippocampal gyrus, *Un* uncus, *FuG* fusiform gyrus, *TP* temporal pole, *a-* anterior, *STG* superior temporal gyrus, *MTG* middle temporal gyrus, *ITG* inferior temporal gyrus, *HS* hippocampal sclerosis, *FCD* focal cortical dysplasia.

**Table 2:** Clinical profiles (Wechsler Memory Scale-Revised).

Case #	Sex/age	WMS-R	Verbal memory	Visual memory	General memory	Attention/concentration	Delayed recall
1	F/27		82	99	85	89	85
2	M/62		84	99	87	106	64
3	M/44		75	106	83	110	81
4	M/40		58	92	63	78	74
5	F/44		87	108	92	92	91
6	M/30		76	111	98	124	84
7	M/19		50	91	50	90	50
8	F/19		65	109	72	118	73
9	M/56		85	95	86	90	86

*F* female, *M* male, *WMS-R* Wechsler Memory Scale-Revised.

### Interictal FC of patients with MTLE compared with that of healthy controls

**Figure 2:**
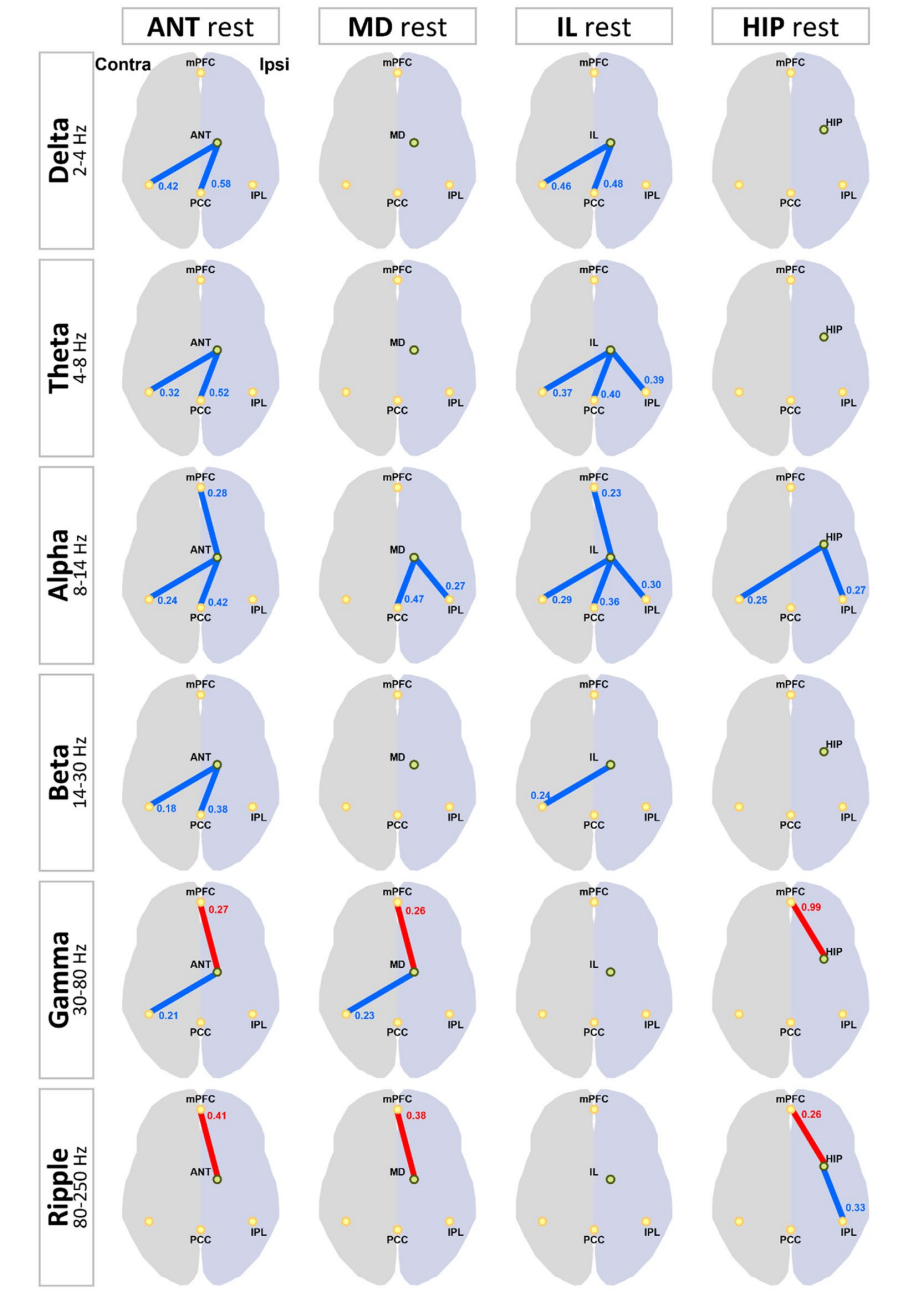
Changes in FC between the thalamic nucleus, hippocampus, and default mode network in patients with mesial temporal lobe epilepsy compared with the FC of healthy controls at the resting state. The changes in FC between the ANT, MD, IL, HIP, and default mode network core system are shown. For each frequency band from the delta to ripple bands, the resting-state FC in the patient with mesial temporal lobe epilepsy (MTLE) is compared with the FC in the healthy controls and shows statistically significant changes; significant increases in FC are indicated by red lines and decreases by blue lines. The number beside the line is the average value of all FC in patients with MTLE. mPFC, medial prefrontal cortex; IPL, inferior parietal lobule; PCC, posterior cingulate cortex; FC, functional connectivity; ANT, anterior nucleus; MD, mediodorsal nucleus; IL, intralaminar nuclei; HIP, hippocampus.

The results of changes in FC between the thalamic nucleus, hippocampus, and DMNRA in patients with MTLE and healthy controls are presented in Supplementary Fig. S1 and are illustrated in Fig. 2. During the interictal periods, the FC between the three thalamic nuclei and the DMNRA of patients with MTLE showed different changes in each frequency band compared with that of healthy controls. In the gamma and ripple bands, there was a significant increase in the FC between the hippocampus (gamma: t = 2.234, *P* = 0.040; ripple: t = 2.611, *P* = 0.019), ANT (gamma: t = 4.600, *P* < 0.001; ripple: t = 3.038, *P* = 0.008), and MD (gamma: t = 5.198, *P* < 0.001; ripple: t = 2.758, *P* = 0.014), which have strong anatomical connections with the hippocampus and mPFC. In contrast, there was no change in the FC between the IL (gamma: t = 1.97, *P* = 0.066; ripple: t = 1.93, *P* = 0.072), which have weak connections with the hippocampus and mPFC in the same bands. The FC between the ANT, IL, and DMNRA broadly decreased in the delta to beta bands, but the changes in FC between the MD, hippocampus, and DMNRA in the same bands were limited. Supplementary Table S2 summarizes all t and *P* values of FC changes with significant differences.

### IED-related FC changes in patients with MTLE

**Figure 3:**
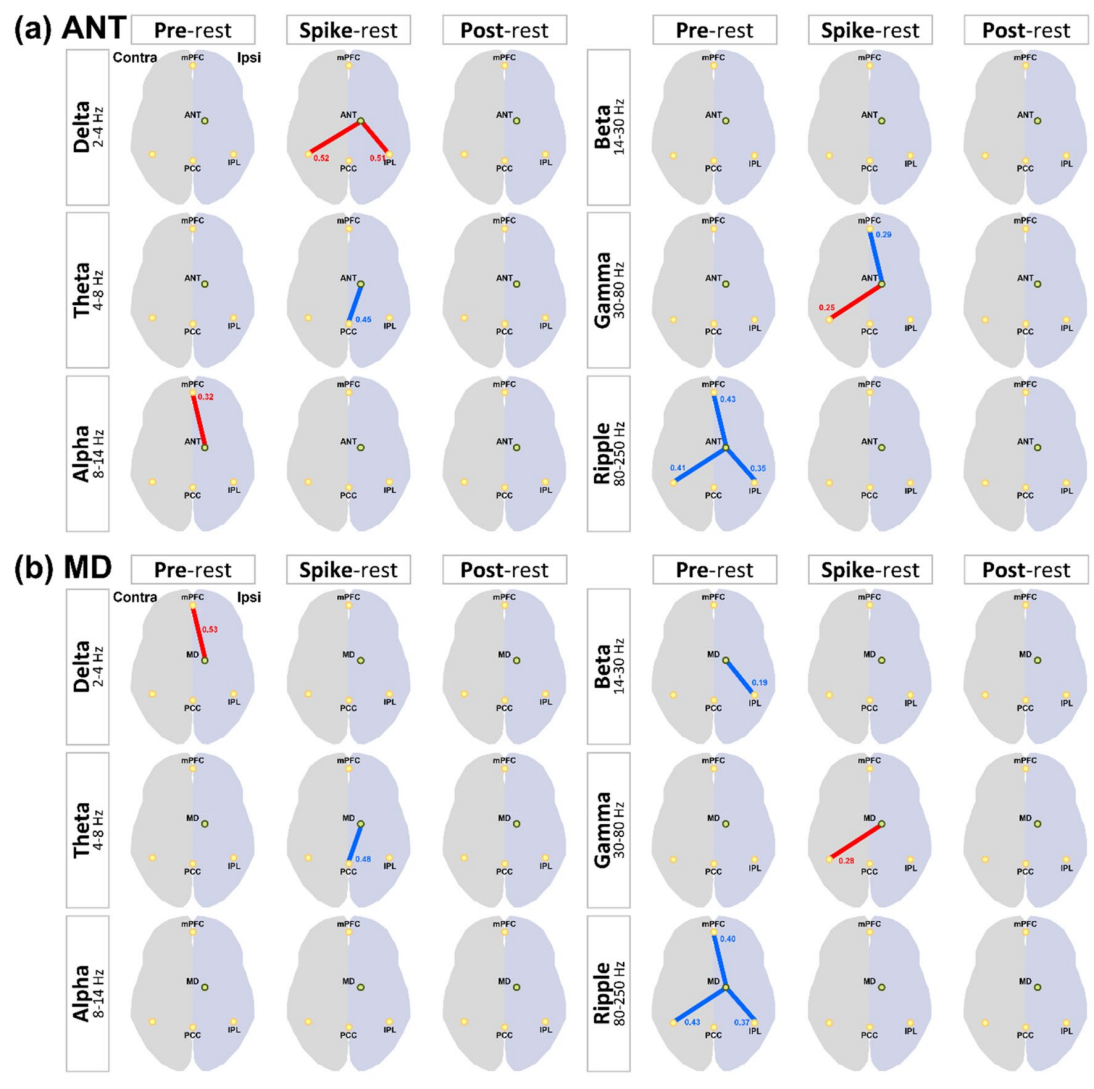

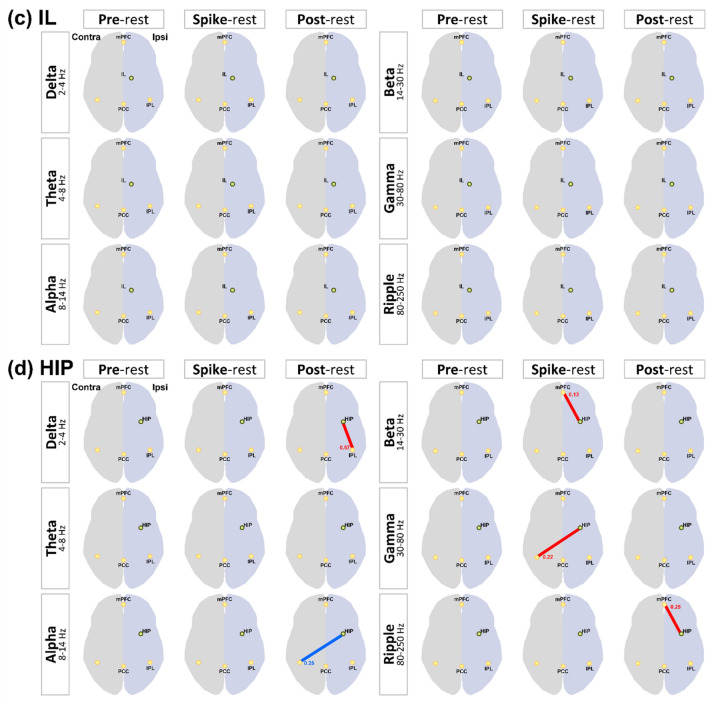
Changes in interictal epileptic discharge-related functional connectivity between the thalamic nucleus, hippocampus, and default mode network in patients with mesial temporal lobe epilepsy. The changes in FC between the (**a**) ANT, (**b**) MD, (**c**) IL, (**d**) HIP, and default mode network core system are shown. For each frequency band from the delta to ripple bands, the FC in the pre-spike, spike, and post-spike periods is compared with the FC in the resting period and shows statistically significant changes; significant increases in FC are indicated by red lines and decreases by blue lines. The number beside the line is the average value of the FC. mPFC, medial prefrontal cortex; IPL, inferior parietal lobule; PCC, posterior cingulate cortex; FC, functional connectivity; ANT, anterior nucleus; MD, mediodorsal nucleus; IL, intralaminar nuclei; HIP, hippocampus.

The results of changes in FC between the thalamic nucleus, hippocampus, and DMNRA associated with IED are shown in Supplementary Figs. S2, S3, and S4 and are illustrated in Fig. 3. Before, during, and after IEDs, significant changes in the FC between the hippocampus, ANT, and MD, which have strong anatomical connections with the hippocampus and DMNRA, were observed compared with that in the resting state. In contrast, the FC between the IL, which have weak anatomical connections with the hippocampus and DMNRA, did not change (Fig. 3c). The changes in the FC between the ANT, MD, and DMNRA started before the onset of a spike and continued during the spike. However, the changes in the FC between the hippocampus and DMNRA were not observed before a spike but started at spike onset and continued after a spike (Fig. 3a-d). In particular, the ANT and MD showed a widespread reduction of FC with the DMNRA in the neural activity of the ripple band in the pre-spike period (Fig. 3a,b). The FC between the hippocampus and DMNRA was elevated in the beta to gamma bands during IEDs and in the delta and ripple bands after IEDs (Fig. 3d). In the gamma band during a spike, the FC between the ANT (t = 2.634, *P* = 0.030), MD (t = 2.841, *P* = 0.022), and hippocampus (t = 2.329, *P* = 0.048) was commonly enhanced with the contralateral IPL (Fig. 3a-d). Supplementary Table S3 shows all t and *P* values of FC changes with significant differences.

## Discussion

The analysis of FC changes associated with IEDs showed that in the pre-spike period before the appearance of IEDs, there was an overall decrease in FC between the ANT and MD, which are strongly connected to the hippocampus, and DMNRA in the ripple band. In contrast, no change was observed in the IL, which are closely connected to the brainstem reticular formation. Changes in the FC between the hippocampus and DMNRA were not observed before but were observed after the start of IEDs (Fig. 3).

Physiological neural activity in the ripple band of the hippocampus is involved in memory consolidation by extensively activating the cortex and inhibiting the brainstem^12^. In a rat model of epilepsy induced by kainic acid administration, abnormal ripple-band neural activity was observed in remote areas, such as the prefrontal cortex, thalamus, and contralateral hippocampus, indicating that the acquisition of epileptogenicity involved the acquisition of a large abnormal network, which was strongly associated with neural activity in the ripple band^13^. Considering these reports, the current results suggest that the neural activity of the normal network in the ripple band at rest was impaired by the abnormal network coupled with the IEDs in patients with MTLE.

Chiosa et al.^15^ reported a 256-channel EEG recording in patients with MTLE. They reported that the FC between the thalamic nucleus and cortex changed before the onset of IEDs, suggesting the role of the thalamus as a pacemaker. Considering these reports, our findings suggested that prior to the onset of IEDs, the ANT and MD were involved in the onset of IEDs, probably working as hubs on different networks.

The ANT and hippocampus form a network connection with the mPFC in addition to the conventional Papez circuit, according to a recent report^7^. The MD is connected to the mPFC and amygdala, especially through the Yakovlev circuit, and the hippocampus through the entorhinal cortex^16^. Therefore, the ANT and MD may regulate the FC between the hippocampus and mPFC at various stages of information processing and may be involved in focusing on a subject during memory processing^8^.

The coupling of neural activity in multiple frequency bands is important in the hippocampal-based memory process, particularly involving activity in the theta, gamma, and ripple bands^11^. Chen et al.^17^ compared diffusion tensor imaging-derived diffusion metrics of ANT in patients with memory impairment due to hydrocephalus with those in healthy controls and found that the FC between the ANT and cortical areas increased in patients with hydrocephalus, reflecting a compensatory mechanism for memory. In this study, a comparison of the resting-state FC of patients with MTLE with that of healthy controls showed that the FC between the ANT, MD, and hippocampus and mPFC were commonly elevated in the gamma to ripple bands (Fig. 2). In this study, patients with MTLE with memory impairment were recruited based on their WMS-R scores and speech therapist diagnoses. The recruited patients with MTLE had lower general memory scores, including verbal memory, on the WMS-R. Multiple comparisons revealed that visual memory and attention/concentration scores were significantly higher than general and verbal memory scores (Table 2). No correlation was found between the FC values and WMS-R sub-items. The reason for this may be the small number of cases, differences in the duration of the illness, level of memory function prior to illness, pathology, and effects of compensatory memory functions, resulting in variability in the degree of impairment in the memory network. Increased FC between the ANT, MD, and hippocampus and mPFC in gamma and ripple bands may suggest that in patients with MTLE, the ANT and MD are involved in neural activity in gamma and ripple bands between the hippocampus and mPFC, a memory network. The mPFC is generally considered to engage in self-reflection, similarly to the DMN, or to be involved in mentalizing. However, this study found no correlation between elevated FC and higher brain function in the mPFC. Therefore, we only confirmed the existence of the elevated FC phenomenon, and future studies are needed to elucidate its relationship with higher brain function.

Tyvaert et al.^18^ showed that the ANT, centromedian nucleus, and parafascicular nucleus included in IL are activated during generalized spike and wave discharges and that the centromedian nucleus and parafascicular nucleus are involved in the initiation or early propagation of IEDs, while the ANT might play a role in maintenance. In the present study, the FC between the ANT, IL, and DMNRA extensively decreased in the delta to beta bands in the resting state in patients with MTLE compared with that in healthy controls (Fig. 2). The pattern of reduced FC between the ANT, IL, and DMNRA is similar, perhaps suggesting that there is a network connection between the thalamic nuclei in the ANT and IL. Although the ANT is a part of the Papez circuit that includes the hippocampus, and the MD is also closely connected to the hippocampus through the Yakovlev circuit, there were no widespread FC changes observed in the hippocampus or MD nucleus similar to those observed between the ANT, IL, and DMNRA. The decrease in the FC between the ANT, IL, and DMNRA suggests that the IL-ANT-cortical circuitry may be affected by the abnormal network of epilepsy. However, this study failed to show an association between these FC changes and higher brain function, which requires further investigation.

This study has some limitations. We only selected patients with MTLE with Engel class I whose seizures resolved after surgical resection of the medial temporal lobe or anterior temporal lobe, considering that these patients had only unilateral network damage centered on the affected side’s medial temporal lobe. In addition, the network damage caused by MTLE may spread over time, and the degree of network damage on the healthy side may vary greatly from patient to patient, depending on the disease duration. Hence, we focused our evaluation on examining the FC between the thalamic nuclei, hippocampus, and DMNRA of the affected side, which had a network disturbance.

Evaluation of deep brain structures using MEG is difficult owing to the rapidly decreasing signal-to-noise ratio^19^. The spatial resolution of subcortical structures, in particular, remains controversial^20^. However, these MEG characteristics do not indicate that it is impossible to observe deep neural activity but only that the sensitivity decreases with depth. Subcortical structures (including the insular gyrus, thalamus, hippocampus, amygdala, and brainstem) can be observed by optimizing the signal extraction method^21^. There have been reports of MEG analysis of the thalamus^22,23^. In a previous study, we adopted the volume head model as a forward model, which weights the dipoles in the space other than the cortical surface as unconstrained ones, without specifying the vectors shown by the dipoles as current sources, instead of the conventional cortical surface modelk^14^. Most of the activity recorded with MEG and EEG comes from the cerebral cortex. However, deep brain structures, such as the limbic system and basal ganglia, contribute to the magnetic fields detected using MEG^24^. The volume head model used in the present study was one of the typical methods for estimating three orthogonal dipoles at each point in the brain. The volume head model was a new source estimation model for MEG that could be used to analyze subcortical function^25–27^. In addition, we used low-resolution brain electromagnetic tomography (sLORETA) as an inverse model, and statistically analyzed its data to show that the focus diagnosis of deep-seated lesions was more accurate than that of conventional methods^14^. In this study, we applied that method to evaluate the FC between the thalamic nucleus and hippocampus and cortex.

The thalamus is not a tissue structure with neurons arranged in rows as those in the cerebral cortex but has a random or spherical arrangement of neurons. Therefore, magnetic fields cancel each other, forming a closed magnetic field that does not allow the magnetic fields to extend outward. Therefore, MEG might not record neural activity in the thalamus. Pizzo et al. reported correlations between stereoelectroencephalography and MEG neural activity in the thalamus from independent component analysis using stereoelectroencephalography electrodes implanted in patients with drug-resistant epilepsy and simultaneously obtained MEG recordings^24^. The thalamus and deep structures, such as the subthalamic nucleus^28^ and basal ganglia^29^, can be analyzed for current sources using MEG. Several studies have reported on MEG analysis using distributed source analysis, including sLORETA for the thalamus, which are listed in Supplementary Table S4. Several reports on detecting thalamic activity using MEG suggest that the closed magnetic field of the thalamus is detectable using MEG or certain parts of the thalamus form an open magnetic field. Attal et al. pointed out that neural generators in deep brain structures are classified as open and closed field cells based on the resulting electromagnetic field produced by their dendritic arborization^30^. This means that the thalamus contains open field cells that can be the source of the current and can be recorded using MEG. The thalamus has previously been analyzed with the whole thalamus as a region of interest using MEG, and no reports exist on the detailed examination of the thalamus at the level of the thalamic nuclei (Supplementary Table S4). This is the first paper to report differential FC with MEG for the thalamic nuclei (ANT, MD, and IL). However, it did not confirm that the neural activity of each thalamic nucleus can be distinguished with MEG recordings in combination with stereoelectroencephalography. Therefore, the accuracy of the neural activity of thalamic nuclei derived from MEG analysis with sLORETA requires further investigation.

In this study, we analyzed FC changes before and after IEDs and between patients with MTLE and healthy controls under matched conditions. However, owing to the small number of cases and data obtained only under limited clinical conditions, making a direct reference to the relationship between FC changes and memory function in some cases was impossible. Future studies are planned to clarify the direct relationship between these FC changes, the frequency of IEDs. and memory function.

Although studies have used EEG and fMRI to examine the FC of the thalamic nucleus and DMN, EEG lacks spatial resolution, whereas fMRI lacks temporal resolution. These methods may only show an indirect correlation because of neurovascular coupling^15,31^. As the neural activities of the thalamic nucleus are involved in memory processes and the hippocampus and DMN are coupled in a specific frequency band, evaluating the FC between the thalamic nucleus, hippocampus, and DMNRA for each frequency band separately is necessary. The present study was the first to use MEG to reveal the FC between the thalamic nucleus, hippocampus, and DMNRA in each frequency band and the FC changes specific to patients with MTLE before and after IEDs and in the resting state compared with the FC of healthy controls.

## Conclusion

We analyzed changes in FC between the thalamic nuclei, hippocampus, and DMNRA, which are involved in memory processes, in each frequency band in patients with MTLE who achieved seizure-free status after surgery for epilepsy. By comparing the FC before and after IEDs and in the resting state and the FC of patients with MTLE with that of healthy controls, we identified FC changes suggestive of network disturbances specific to patients with MTLE. The reduced FC between the ANT and MD, which are closely connected to the hippocampus, and the DMNRA in the period just before the IEDs in the ripple band suggested the presence of an abnormal network associated with IEDs. Comparisons between patients with MTLE and healthy controls in the interictal period revealed increased FC in the gamma to ripple bands between the hippocampus and between the ANT and MD connecting to the hippocampus and mPFC and similar widespread FC decrease in the delta to beta bands between the ANT, IL, and DMNRA. These FC changes between thalamic nuclei involved in memory, the hippocampus, and DMNRA may be related to memory impairment in patients with MTLE.

## Methods

### Participants

We retrospectively evaluated 12 consecutive patients aged over 18 years who underwent preoperative MEG, the WMS-R^32^ scoring by a speech therapist, and lesionectomy, including standard temporal lobectomy and selective amygdalohippocampectomy, at our institute between October 2014 and December 2019. Patients were hospitalized for 1 week and underwent a comprehensive neuropsychological evaluation. The rehabilitation doctor selected the necessary examination for the patient and evaluated the final results. The speech therapist obtained the medical history, performed the neuropsychological examination, evaluated the scores, and diagnosed the presence or absence of memory impairment, and the diagnosis was approved by rehabilitation doctors. To maintain the objectivity of the neuropsychological examination, the neurosurgeon was not involved in any part of the examination process and was only informed of the results of the diagnosis. Of the 12 eligible patients, three were excluded because their postoperative seizure outcomes during 2 years after surgery^33^ were poor, and they were evaluated as having class II, III, or IV in Engel’s classification. Nine patients with preoperative memory impairment diagnosed by a speech therapist to be at Engel class I and free of disabling seizures and nine age- and sex-matched healthy controls were included in the FC analysis. Healthy controls were recruited from the Nagoya University healthy cohort^34^. This cohort was assessed, and dementia (cutoff: 88/89) and mild cognitive impairment (cutoff: 82/83) were ruled out using the Addenbrooke's Cognitive Examination^35^. Multiple comparisons (Tukey’s honestly significant difference test) were performed between WMS-R sub-items (including verbal memory, visual memory, general memory, attention/concentration, and delayed recall) in patients with MTLE using IBM SPSS Statistics (International Business Machines Corporation, Armonk, NY, USA). Statistical significance was set at *P* < 0.05.

The ethics committee of Nagoya University Graduate School of Medicine approved this study (No. 1005-2). Informed written consent was obtained from the patients and their families. All methods were performed in accordance with the relevant guidelines and regulations.

### MEG

Magnetic signals on interictal periods in stage one to two of the sleep state were recorded for each participant using a whole-head MEG system with 160 axial gradiometers (PQ1160C, Ricoh Corp., Tokyo, Japan). No drug was used to induce sleep. Magnetic responses were filtered using a 1–2000 Hz initial band-pass filter and digitized with a sampling frequency of 5,000 Hz. EEG at eight scalp areas (F3, F4, C3, C4, T3, T4, P3, and P4) based on the international 10–20 system and electrocardiography was simultaneously performed with a 1–100-Hz bandpass filter at the same sampling rate. The participant's head information was coregistered using standard reference points, head position indicator coils, and 3D-digitizer scans of the scalp surface. Each participant completed five recording sessions of 4 min each. The MEG and EEG signals were visually observed on the monitors in real time during the recording. For patients with MTLE, when IEDs were detected on the monitor more than 10 times, i.e., when they were sufficient for further analyses, the recordings were completed in five sessions (20 min total); otherwise, the recordings were extended for up to seven recording sessions (28 min total) until 10 IEDs were detected on the monitor.

### MRI data preprocessing and head model construction

MRI T1-weighted sagittal sections (slice thickness, 1.0 mm; echo time, 2.5 ms; repetition time, 2500 ms; 192 slices) were obtained using a 3 T MR scanner (Siemens, Munich, Germany; 3 Tesla System). We processed the brain structure extraction from MRI using the BrainSuite open-source software (Signal and Image Processing Institute, Department of Electrical Engineering Systems, University of Southern California)^36^. Individual MRI data were realigned and normalized to the Montreal Neurological Institute (MNI) space. The computed head model was developed based on the processed data using the Brainstorm open-source software from the same institute^37^. We used the standard template, ICBM152^38^, for all patients and healthy controls as the source space and overlapping spheres and the volume head model for the forward modelling^14,39^. The volume head model used in the present study has been one of the typical methods to estimate three orthogonal dipoles at each point in the brain. The volume head model was a new source estimation model for MEG that was used to analyze subcortical function^25–27^. The volume head model was calculated by placing the current source on a 5 mm isotropic grid in the brain parenchyma, and the estimated grid location number in the parenchyma was 13,478. We confirmed that the coordinates of the three thalamic nuclei used in this study were computed from separate grids on the Brainstorm software.

### MEG data preprocessing

The recorded MEG data were preprocessed using the Brainstorm software. The MEG recordings obtained during sleep stage one to two were considered the object of the analyses. In patients with MTLE, the IEDs that were initially identified and collected from MEG recordings were visually inspected by three epilepsy specialists (TI, SM, and YH). IED spike onset and offset times were recorded in MEG sensor space. Reproducible spikes were chosen for analysis on unanimous agreement. However, non-reproducible spike-like activities that appeared only once in the recording were excluded only when they were judged to be nonepileptic upon unanimous agreement. All MEG data were divided into six bands: delta (2–4 Hz), theta (4–8 Hz), alpha (8–14 Hz), beta (14–30 Hz), gamma (30–80 Hz), and ripple (80–250 Hz) using a bandpass filter. We used sLORETA^40^ as the inverse model. sLORETA was set to an unconstrained dipole orientation, and the noise covariance regularization was set to a diagonal noise covariance. The regularization parameter was set to three as the signal-to-noise ratio. The output mode was inverse kernel only.

### FC and seed regions

We defined the MNI coordinates of the seed regions, which were the three thalamic nuclei (ANT, MD, and IL) of the affected side, affected side of the hippocampus, and DMN core system (DMNRA, mPFC, IPL, and PCC), referencing the stereotactic atlases^41,42^ and past reports^43–49^. The MNI coordinates of the seed regions are presented in Table 3. In this study, we converted the affected side of patients with MTLE and the same side of the matched healthy controls to the left side. As we hypothesized that the FC between the thalamic nuclei, hippocampus, and DMNRA would vary across frequency bands, we evaluated FC using magnitude-square coherence, which measures the covariance of the two signals in the frequency domain, rather than solely relying on amplitude, as correlation analyses does. Seed region configuration and FC calculations were performed using Brainstorm software.

**Table 3:** Montreal Neurological Institute coordinates of seed regions.

Regions		MNI coordinates (x, y, z, mm) (Ipsi = left, Contra = right)
Thalamus	Anterior nucleus	Ipsi = − 6, − 8, 14
	Mediodorsal nucleus	Ipsi = − 4, − 13, 9
	Intralaminar nuclei	Ipsi = − 11, − 21, 1
Hippocampus		Ipsi = − 29, − 19, − 15
Default mode network core system	Medial prefrontal cortex	− 1, 54, 27
	Inferior parietal lobule	Ipsi = − 46, − 66, 30; Contra = 49, − 63, 33
	Posterior cingulate cortex	0, − 52, 27

Affected side of patients with mesial temporal lobe epilepsy and the same side of the matched healthy controls were converted to the left side.

*MNI* Montreal Neurological Institute, *Ipsi* ipsilateral (left, affected side), *Contra* contralateral (right, healthy side).

### Interictal FC of patients with MTLE compared with that of healthy controls

Three second interictal periods in sleep stage one to two, not including the period from 3 s before to 6 s after the IED in patients with MTLE, were cut out at 50 time points from MTLE and healthy MEG recordings. The FC was analyzed among the affected-side thalamic nuclei, hippocampus, and DMN core system (DMNRA) in each of the six frequency bands (delta, theta, alpha, beta, gamma, and ripple) and averaged for each patient and control using Brainstorm software. Multiple comparisons among the FC are inappropriate, as each FC between the thalamic nuclei, hippocampus, and DMNRA is a part of a different functional connectome; precisely, these FCs do not belong to the same population. Therefore, a comparison between two groups (MTLEs vs. healthy controls) at each connection is an appropriate statistical approach. Therefore, in this study, changes in each network during the spike periods to the resting state were evaluated for each frequency band. For this reason, significant changes in the FC between the patients with MTLE and healthy controls were evaluated with a two-sample t-test using IBM SPSS Statistics (IBM Corp.), and *P* < 0.05 indicated significance.

### IED-related FC changes in patients with MTLE

We defined four 3 s periods based on IED spike onset (0 s): rest (− 6 to − 3 s), pre-spike (− 3 to 0 s), spike (0 to 3 s), and post-spike (3 to 6 s), with no previous spike and noise in the preceding 12 s from MEG recordings of patients with MTLE. In total, 53 IEDs (average 6.6 IEDs per patient) were analyzed in eight patients with MTLE. The FC was analyzed among the affected-side thalamic nuclei, hippocampus, and DMN core system in each of the six frequency bands (delta, theta, alpha, beta, gamma, and ripple) and averaged for each patient using Brainstorm software. As it is not appropriate to make multiple comparisons between each network examined in this study for the same reasons as those for the resting-state FC analysis described above, the FC of the pre-spike, spike, and post-spike periods were compared with the FC of the resting period, and significant changes were assessed with a paired t-test using IBM SPSS Statistics (IBM Corp.), with *P* < 0.05 indicating significance.

## Supplementary Information

Supplementary Information.

## Data Availability

The datasets generated during and/or analyzed during the current study are available from the corresponding author on reasonable request.
