# Supplementary material for: Connectivity alteration in thalamic nuclei and default mode network-related area in memory processes in mesial temporal lobe epilepsy using magnetoencephalography
Source: Sci Rep. 2023 Jun 30;13:10632. doi: 10.1038/s41598-023-37834-2 (PMC10313774; doi:10.1038/s41598-023-37834-2)
Supplement: Supplementary file 1 — Supplementary Information. [file 41598_2023_37834_MOESM1_ESM.pdf]

Connectivity alteration in thalamic nuclei and default mode network-related area in memory processes in mesial temporal lobe epilepsy using magnetoencephalography

Tomotaka Ishizaki[a], Satoshi Maesawa[a,b*], Daisuke Nakatsubo[a,b], Hiroyuki Yamamoto[b,c], Jun Torii[a], Manabu Mutoh[a], Jun Natsume[b,c], Minoru Hoshiyama[b], Ryuta Saito[a]

[a]Department of Neurosurgery, Nagoya University Graduate School of Medicine, Nagoya, Aichi, Japan
[b]Brain and Mind Research Center, Nagoya University, Nagoya, Aichi, Japan
[c]Department of Pediatrics, Nagoya University Graduate School of Medicine, Nagoya, Aichi, Japan

CORRESPONDING AUTHOR:
Satoshi Maesawa
Department of Neurosurgery, Nagoya University Graduate School of Medicine
Nagoya, Aichi, Japan, 65 Tsurumai, Showa, Nagoya, Aichi, 466-8550, Japan
Tel.: +81(52)744-2353
Fax: +81(52)744-2360
E-mail address: smaesawa@med.nagoya-u.ac.jp

Supplementary Table 1. Clinical profiles of healthy controls

Healthy controls	Sex/Age	ACE-R scores					
		Total (100)	Attention/ Orientation (18)	Fluency (14)	Memory (26)	Language (26)	Visospatial (16)
1	F/27	95	18	14	24	25	14
2	M/62	97	18	14	25	25	15
3	M/43	98	18	14	24	26	16
4	M/40	98	18	14	25	25	16
5	F/44	99	18	14	26	25	16
6	M/30	98	18	14	24	26	16
7	M/21	95	18	14	23	24	16
8	F/20	95	18	14	23	24	16
9	M/56	100	18	14	26	26	16

F, female; M, male; ACE-R, Addenbrooke's Cognitive Examination.

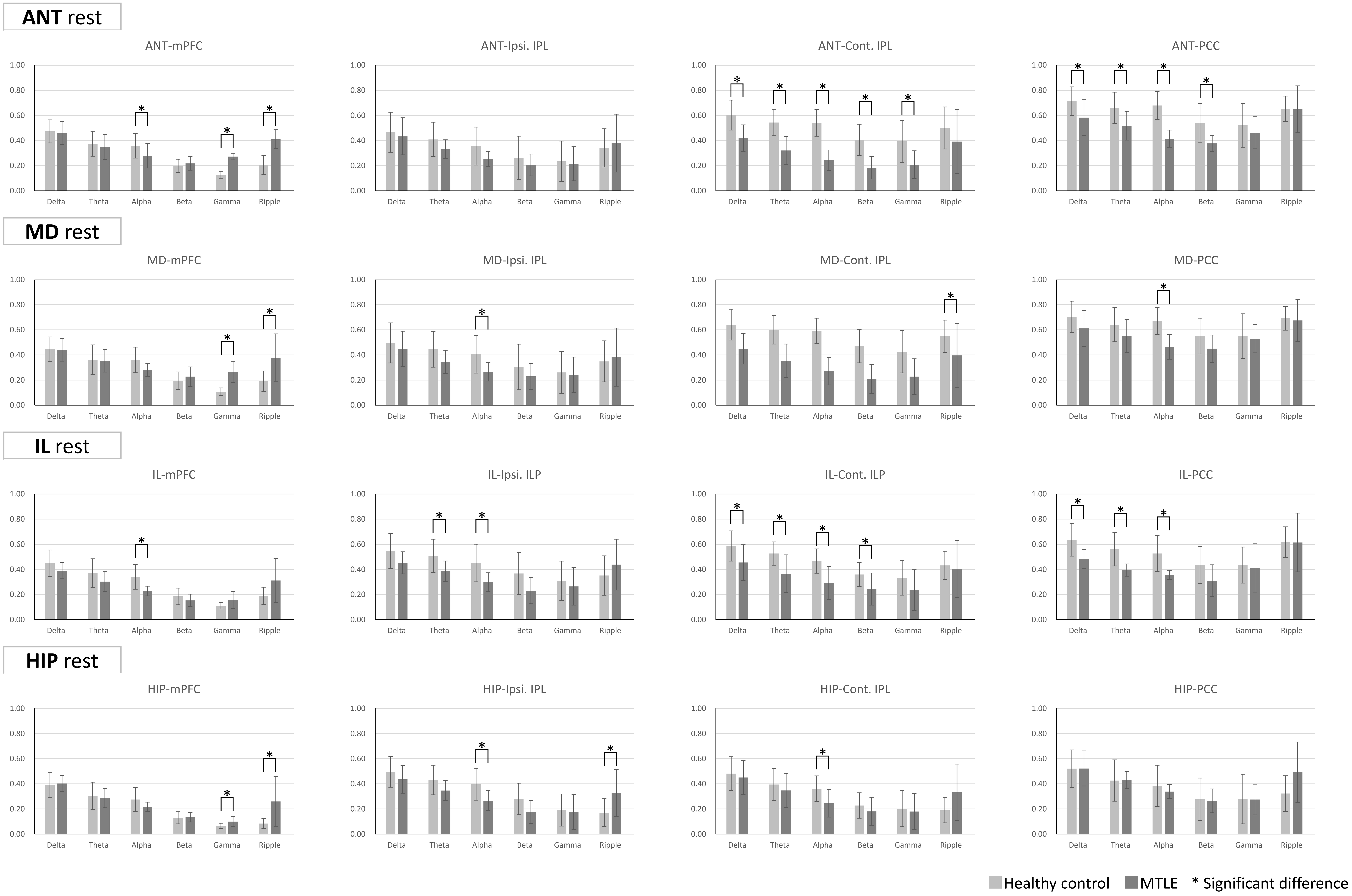

Supplementary Figure 1. Changes in functional connectivity between the thalamic nuclei, hippocampus, and default mode network in a patient with mesial temporal lobe epilepsy compared with that in healthy controls at the resting state

The changes in functional connectivity (FC) between the anterior nucleus (ANT), mediodorsal nucleus (MD), intralaminar nuclei (IL), hippocampus (HIP), and default mode network core system (medial prefrontal cortex, mPFC; inferior parietal lobule, IPL; and posterior cingulate cortex, PCC) are shown.

The resting-state FC for each frequency band, ranging from delta to ripple, in the patient with mesial temporal lobe epilepsy (dark grey) is compared with the FC in healthy controls (light grey). An asterisk indicates significant changes.

Supplementary Table 2. Interictal functional connectivity changes with significant differences

Frequency bands	Changed FC	FC change	Average of FC	t-value	P-value
Delta	ANT-PCC	Decrease	0.582	-2.172	0.045
	ANT-Contra IPL	Decrease	0.420	-3.471	0.003
	IL-PCC	Decrease	0.483	-3.062	0.007
	IL-Contra IPL	Decrease	0.455	-2.125	0.049
Theta	ANT-PCC	Decrease	0.518	-2.498	0.024
	ANT-Contra IPL	Decrease	0.322	-4.363	<0.001
	IL-PCC	Decrease	0.395	-3.501	0.003
	IL-Ipsi IPL	Decrease	0.386	-2.343	0.032
	IL-Contra IPL	Decrease	0.366	-2.735	0.015
Alpha	ANT-mPFC	Decrease	0.280	-2.135	0.024
	ANT-PCC	Decrease	0.415	-6.039	<0.001
	ANT-Contra IPL	Decrease	0.244	-6.656	<0.001
	MD-PCC	Decrease	0.465	-4.182	<0.001
	MD-Ipsi IPL	Decrease	0.266	-2.507	0.028
	IL-mPFC	Decrease	0.227	-3.202	0.009
	IL-PCC	Decrease	0.356	-3.472	0.003
	IL-Ipsi IPL	Decrease	0.298	-2.739	0.015
	IL-Contra IPL	Decrease	0.292	-3.181	0.006
	HIP-Ipsi IPL	Decrease	0.266	-2.595	0.020
	HIP-Contra IPL	Decrease	0.245	-2.313	0.034
Beta	ANT-PCC	Decrease	0.377	-2.955	0.009
	ANT-Contra IPL	Decrease	0.183	-4.356	<0.001
	IL-Contra IPL	Decrease	0.243	-2.186	0.044
Gamma	ANT-mPFC	Increase	0.272	4.600	<0.001
	ANT-Contra IPL	Decrease	0.208	-2.799	0.013
	MD-mPFC	Increase	0.264	5.198	<0.001
	MD-Contra IPL	Decrease	0.228	-2.688	0.016
	HIP-mPFC	Increase	0.993	2.234	0.040
Ripple	ANT-mPFC	Increase	0.410	3.038	0.008
	MD-mPFC	Increase	0.379	2.758	0.014
	HIP-mPFC	Increase	0.260	2.611	0.019
	HIP-Ipsi IPL	Decrease	0.326	2.150	0.047

FC, functional connectivity; ANT, anterior nucleus; MD, mediodorsal nucleus; IL, intralaminar nuclei of thalamus; HIP, hippocampus; IPL, inferior parietal lobule; PCC, posterior cingulate cortex; mPFC, medial prefrontal cortex; Ipsi, ipsilateral (left, affected side); Contra, contralateral (right, healthy side).

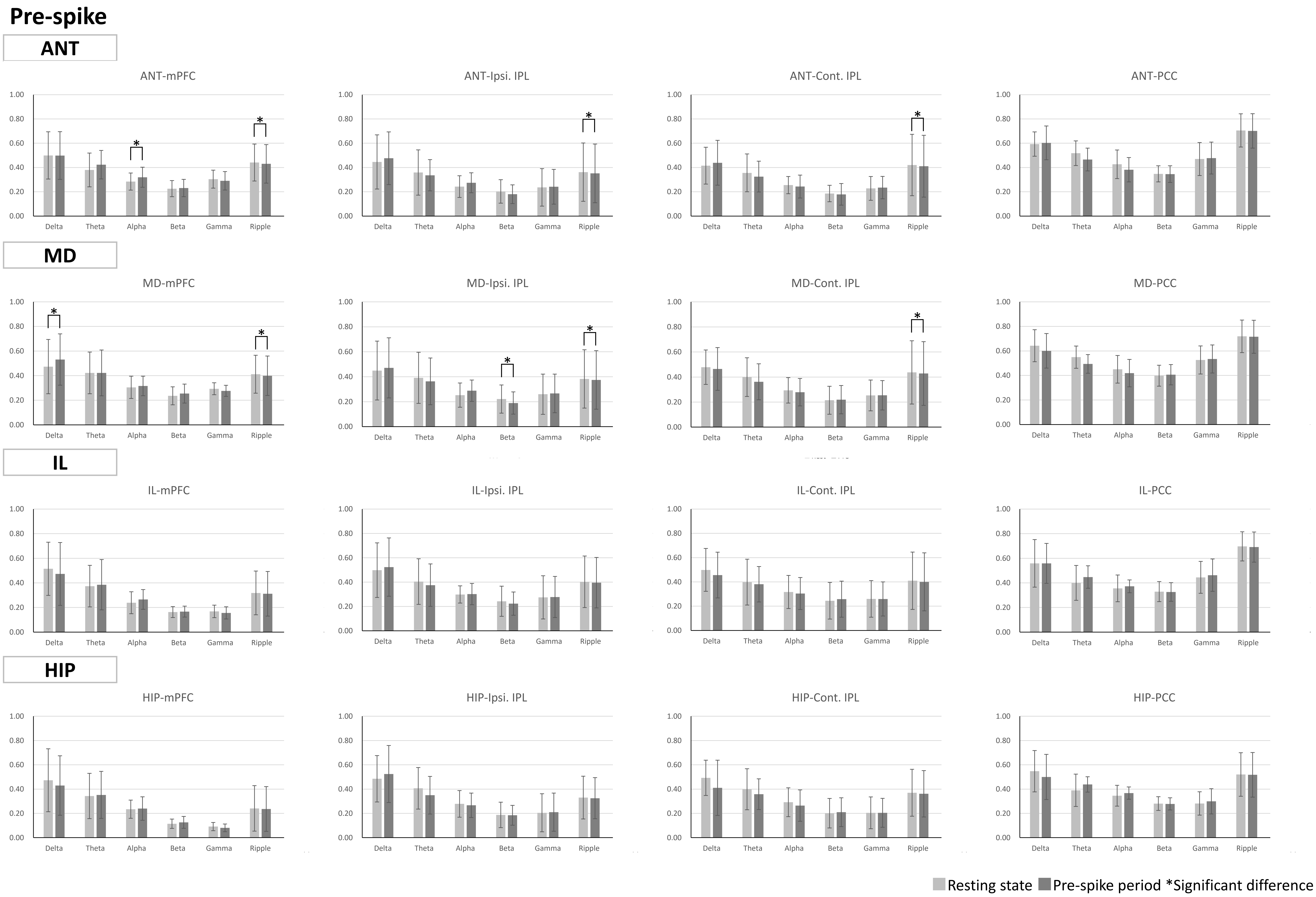

Supplementary Figure 2. Changes in pre-spike-period functional connectivity between the thalamic nuclei, hippocampus, and default mode network in patients with mesial temporal lobe epilepsy

The changes in functional connectivity (FC) between the (A) anterior nucleus (ANT), (B) mediodorsal nucleus (MD), (C) intralaminar nuclei (IL), (D) hippocampus (HIP), and default mode network core system (medial prefrontal cortex, mPFC; inferior parietal lobule, IPL; and posterior cingulate cortex, PCC) are shown.

The FC for each frequency band, ranging from delta to ripple, in the pre-spike periods is compared with the FC in the resting period and shows statistically significant changes using an asterisk.

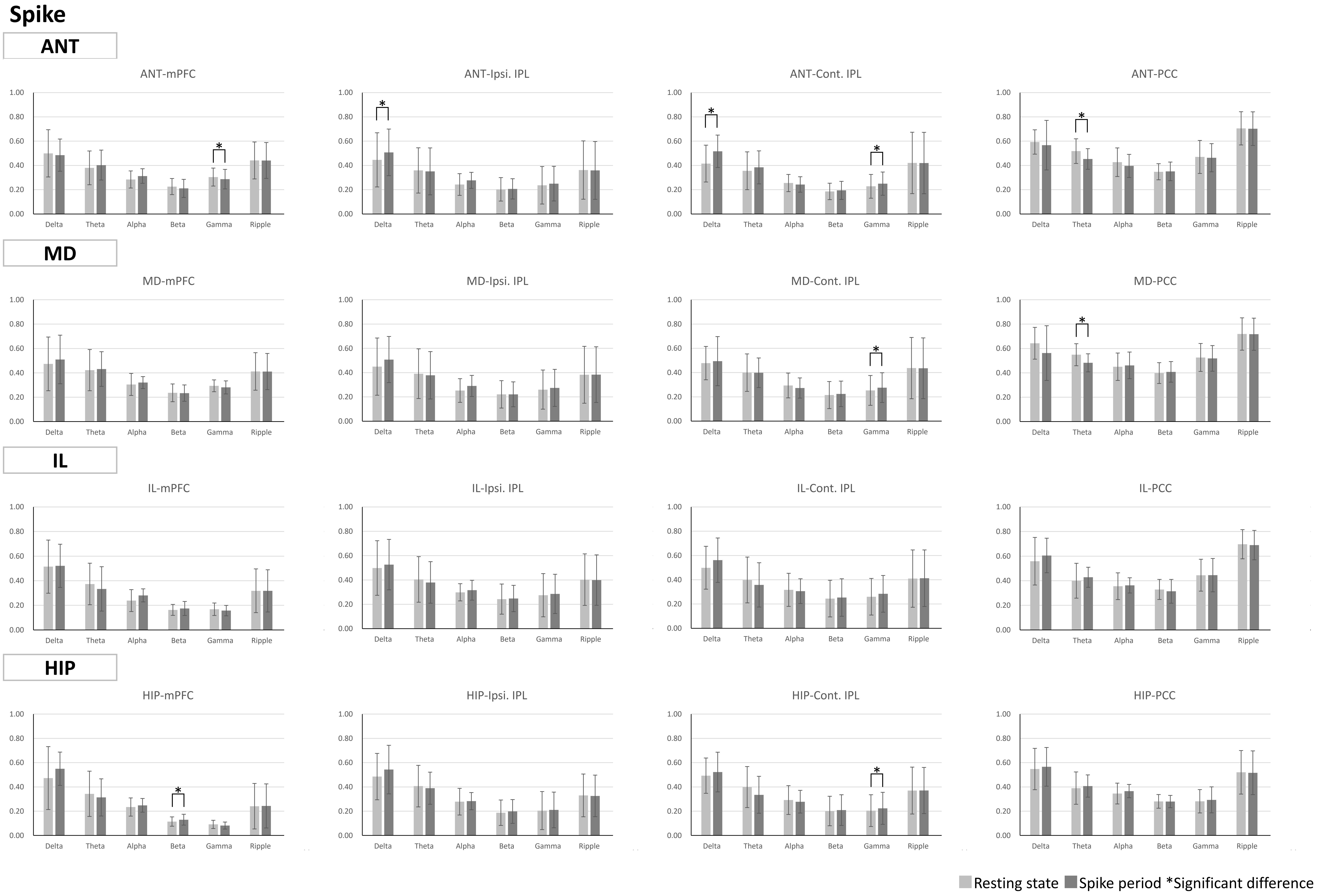

Supplementary Figure 3. Changes in spike-period functional connectivity between the thalamic nuclei, hippocampus, and default mode network in patients with mesial temporal lobe epilepsy

The changes in functional connectivity (FC) between the (A) anterior nucleus (ANT), (B) mediodorsal nucleus (MD), (C) intralaminar nuclei (IL), (D) hippocampus (HIP), and default mode network core system (medial prefrontal cortex, mPFC; inferior parietal lobule, IPL; and posterior cingulate cortex, PCC) are shown.

The FC for each frequency band, ranging from delta to ripple, in the spike periods is compared with the FC in the resting period and shows statistically significant changes using an asterisk.

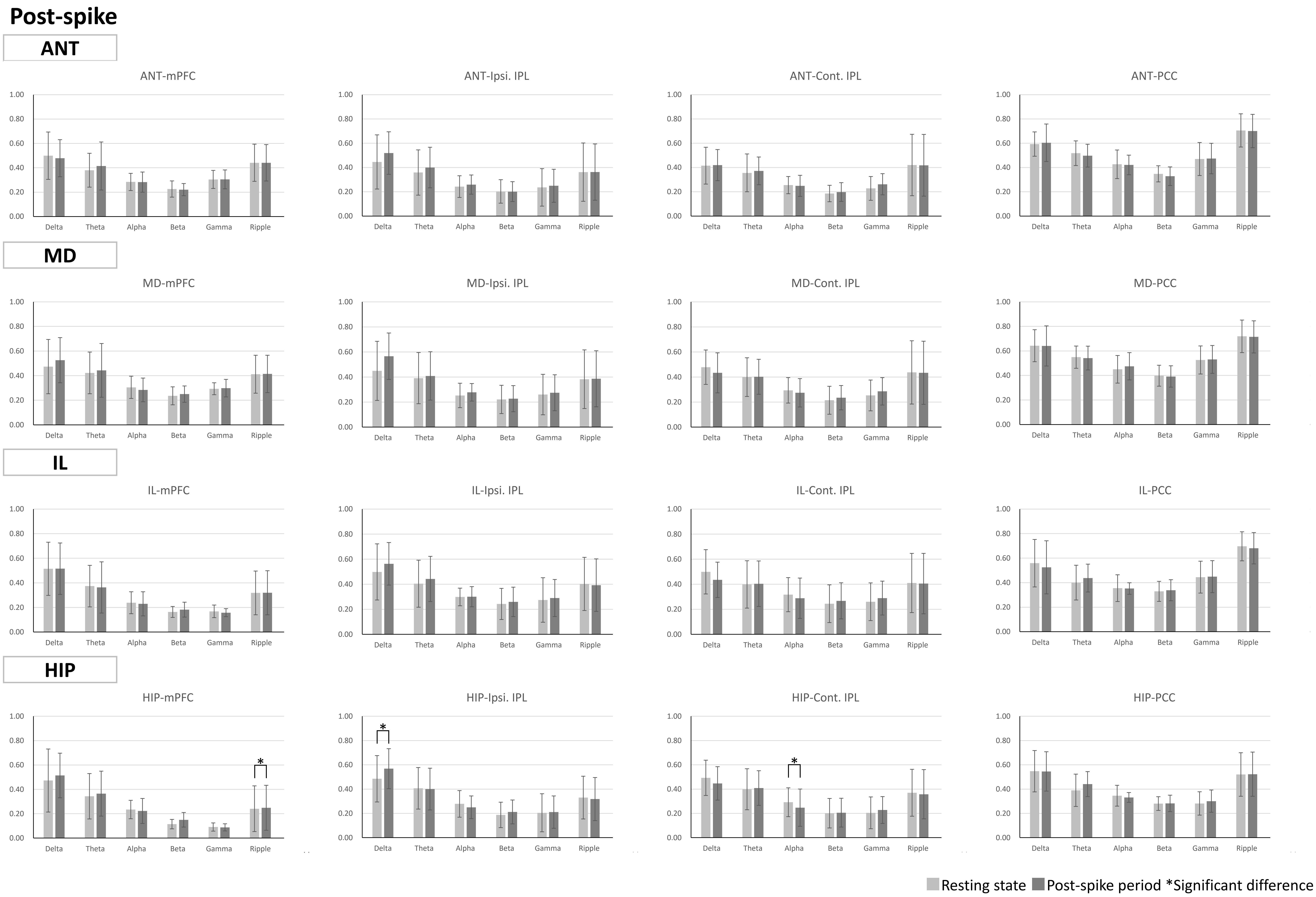

Supplementary Figure 4. Changes in post-spike-period functional connectivity between the thalamic nuclei, hippocampus, and default mode network in patients with mesial temporal lobe epilepsy

The changes in functional connectivity (FC) between the (A) anterior nucleus (ANT), (B) mediodorsal nucleus (MD), (C) intralaminar nuclei (IL), (D) hippocampus (HIP), and default mode network core system (medial prefrontal cortex, mPFC; inferior parietal lobule, IPL; and posterior cingulate cortex, PCC) are shown.

The FC for each frequency band, ranging from delta to ripple, in the post-spike periods is compared with the FC in the resting period and shows statistically significant changes using an asterisk.

Supplementary Table 3. Interictal epileptic discharge-related functional connectivity changes with significant differences

IED periods	Frequency bands	Changed FC	FC change	Average of FC	t-value	P-value
Pre	Delta	MD-mPFC	Increase	0.531	2.719	0.026
	Alpha	ANT-mPFC	Increase	0.320	2.472	0.039
	Beta	MD-Ipsi IPL	Decrease	0.189	-2.569	0.033
	Ripple	ANT-mPFC	Decrease	0.430	-2.573	0.033
		ANT-Ipsi IPL	Decrease	0.359	-2.415	0.042
		ANT-Contra IPL	Decrease	0.411	-3.060	0.016
		MD-mPFC	Decrease	0.399	-3.067	0.015
		MD-Ipsi IPL	Decrease	0.375	-2.408	0.043
		MD-Contra IPL	Decrease	0.428	-2.775	0.024
Spike	Delta	ANT-Ipsi IPL	Increase	0.508	2.400	0.043
		ANT-Contra IPL	Increase	0.517	3.167	0.013
	Theta	ANT-PCC	Decrease	0.453	-2.572	0.033
		MD-PCC	Decrease	0.482	-2.709	0.027
	Beta	HIP-mPFC	Increase	0.130	3.030	0.016
	Gamma	ANT-mPFC	Decrease	0.287	-2.642	0.030
		ANT-Contra IPL	Increase	0.249	2.634	0.030
		MD-Contra IPL	Increase	0.276	2.841	0.022
		HIP-Contra IPL	Increase	0.223	2.329	0.048
Post	Delta	HIP-Ipsi IPL	Increase	0.568	2.849	0.022
	Alpha	HIP-Contra IPL	Decrease	0.247	-2.355	0.046
	Ripple	HIP-mPFC	Increase	0.249	2.453	0.040

IED, interictal epileptic discharge; FC, functional connectivity; ANT, anterior nucleus; MD, mediodorsal nucleus; IL, intralaminar nuclei of thalamus; HIP, hippocampus; IPL, inferior parietal lobule; PCC, posterior cingulate cortex; mPFC, medial prefrontal cortex; Ipsi, ipsilateral (left, affected side); Contra, contralateral (right, healthy side).

Supplementary Table 4. Reports of current source analysis of thalamus and basal ganglia using magnetoencephalography

Author	Year	Journal	Participants, Patients, Subjects	Methods	Inverse modeling	Analysis	Thalamic ROI
Min et al.	2020	Neuroimage	15 HV	MEG	DS (weighted minimum-norm estimate)	EC (dynamic causal modeling)	Whole
Piastra et al.	2020	Human Brain Mapping	HV	MEG, EEG	ECD	Dipole source localization	Whole, caudate, putamen, pallidum
Jiang et al.	2019	Frontiers in Neurology	15 CAE	MEG	DS (accumulated source imaging)	Source localization, EC (Granger causality test)	Whole
Pizzo et al.	2019	Nature Communications	14 focal epilepsy	MEG, SEEG	N/A	Independent component analysis	Whole
Miao et al.	2019	Brain Topography	25 CAE	MEG	DS (beamformer, accumulated source imaging)	FC (correlation)	Whole
van Wijk et al.	2018	Neuroimage	11 Parkinson's disease treated with deep brain stimulation	MEG、Deep brain stimulation	DS (beamformer)	EC (dynamic causal modeling)	Not thalamus; subthalamic nucleus
Youssofzadeh et al.	2018	Epilepsy Research	16 CAE	MEG	DS (LCMV beamformer)	Eigenvector centrality, Phase locking value	Whole
Tenney et al.	2018	Epilepsia	17 CAE	MEG	DS (LCMV beamformer)	EC (phase slope index)	Whole
Wu et al.	2017	Neuroscience	14 CAE	MEG	DS (beamformer, accumulated source imaging)	FC (correlation), EC (Granger causality test)	Whole
Tenney et al.	2013	Epilepsy Research	12 CAE	MEG	DS (sLORETA, beamformer)	Source localization	Whole
Roux et al.	2013	Journal of Neuroscience	45 HV	MEG	DS (linearly constrained minimum variance beamformer)	Phase-amplitude coupling	Whole
Papadelis et al.	2012	Neuroimage	14 HV, 1 patient	MEG	ECD	Dipole source localization	Whole
Ikeda et al.	2002	Journal of Neurophysiology	18, 12 and 5 piglets	MEG	N/A	High-frequency oscillation	Whole
Gobbele et al.	1998	Electroencephalography and clinical Neurophysiology	10 HV	MEG	ECD	Dipole source localization	Whole

Healthy volunteer; HV, Childhood absense epilepsy; CAE, Distributed source analysis; DS, Equivalent source analysis; ECD, Not applicable; N/A, Standardized low resolution brain electromagnetic tomography; sLORETA, Functional connectivity; FC, Effective connectivity; EC.

REFERENCES

1 Min, B. K., Kim, H. S., Pinotsis, D. A. & Pantazis, D. Thalamocortical inhibitory dynamics support conscious perception. *Neuroimage* **220**, 117066 (2020).

2 Piastra, M. C. *et al.* A comprehensive study on electroencephalography and magnetoencephalography sensitivity to cortical and subcortical sources. *Hum. Brain Mapp.* **42**, 978-992 (2021).

3 Jiang, W. *et al.* Dynamic Neuromagnetic Network Changes of Seizure Termination in Absence Epilepsy: A Magnetoencephalography Study. *Front. Neurol.* **10**, 703 (2019).

4 Pizzo, F. *et al.* Deep brain activities can be detected with magnetoencephalography. *Nat Commun* **10**, 971 (2019).

5 Miao, A. *et al.* Ictal Source Locations and Cortico-Thalamic Connectivity in Childhood Absence Epilepsy: Associations with Treatment Response. *Brain Topogr.* **32**, 178-191 (2019).

6 van Wijk, B. C. M., Cagnan, H., Litvak, V., Kuhn, A. A. & Friston, K. J. Generic dynamic causal modelling: An illustrative application to Parkinson's disease. *Neuroimage* **181**, 818-830 (2018).

7 Youssofzadeh, V., Agler, W., Tenney, J. R. & Kadis, D. S. Whole-brain MEG connectivity-based analyses reveals critical hubs in childhood absence epilepsy. *Epilepsy Res.* **145**, 102-109 (2018).

8 Tenney, J. R. *et al.* Ictal connectivity in childhood absence epilepsy: Associations with outcome. *Epilepsia* **59**, 971-981 (2018).

9 Wu, C. *et al.* Quantify neuromagnetic network changes from pre-ictal to ictal activities in absence seizures. *Neuroscience* **357**, 134-144 (2017).

10 Tenney, J. R. *et al.* Focal corticothalamic sources during generalized absence seizures: a MEG study. *Epilepsy Res.* **106**, 113-122 (2013).

11 Roux, F., Wibral, M., Singer, W., Aru, J. & Uhlhaas, P. J. The phase of thalamic alpha activity modulates cortical gamma-band activity: evidence from resting-state MEG recordings. *J. Neurosci.* **33**, 17827-17835 (2013).

12 Papadelis, C., Leonardelli, E., Staudt, M. & Braun, C. Can magnetoencephalography track the afferent information flow along white matter thalamo-cortical fibers? *Neuroimage* **60**, 1092-1105 (2012).

13 Ikeda, H., Leyba, L., Bartolo, A., Wang, Y. & Okada, Y. C. Synchronized spikes of thalamocortical axonal terminals and cortical neurons are detectable outside the pig brain with MEG. *J. Neurophysiol.* **87**, 626-630 (2002).

14 Gobbele, R., Buchner, H. & Curio, G. High-frequency (600 Hz) SEP activities originating in the subcortical and cortical human somatosensory system. *Electroencephalogr. Clin. Neurophysiol.* **108**, 182-189 (1998).
